# The Prediction of Key Cytoskeleton Components Involved in Glomerular Diseases Based on a Protein-Protein Interaction Network

**DOI:** 10.1371/journal.pone.0156024

**Published:** 2016-05-26

**Authors:** Fangrui Ding, Aidi Tan, Wenjun Ju, Xuejuan Li, Shao Li, Jie Ding

**Affiliations:** 1 Department of Pediatrics, Peking University First Hospital, Beijing, China; 2 MOE Key Laboratory of Bioinformatics and Bioinformatics Division, TNLIST, Department of Automation, Tsinghua University, Beijing, China; 3 Department of Internal Medicine, University of Michigan, Ann Arbor, MI, United States of America; University Medical Center Utrecht, NETHERLANDS

## Abstract

Maintenance of the physiological morphologies of different types of cells and tissues is essential for the normal functioning of each system in the human body. Dynamic variations in cell and tissue morphologies depend on accurate adjustments of the cytoskeletal system. The cytoskeletal system in the glomerulus plays a key role in the normal process of kidney filtration. To enhance the understanding of the possible roles of the cytoskeleton in glomerular diseases, we constructed the Glomerular Cytoskeleton Network (GCNet), which shows the protein-protein interaction network in the glomerulus, and identified several possible key cytoskeletal components involved in glomerular diseases. In this study, genes/proteins annotated to the cytoskeleton were detected by Gene Ontology analysis, and glomerulus-enriched genes were selected from nine available glomerular expression datasets. Then, the GCNet was generated by combining these two sets of information. To predict the possible key cytoskeleton components in glomerular diseases, we then examined the common regulation of the genes in GCNet in the context of five glomerular diseases based on their transcriptomic data. As a result, twenty-one cytoskeleton components as potential candidate were highlighted for consistently down- or up-regulating in all five glomerular diseases. And then, these candidates were examined in relation to existing known glomerular diseases and genes to determine their possible functions and interactions. In addition, the mRNA levels of these candidates were also validated in a puromycin aminonucleoside(PAN) induced rat nephropathy model and were also matched with existing Diabetic Nephropathy (DN) transcriptomic data. As a result, there are 15 of 21 candidates in PAN induced nephropathy model were consistent with our predication and also 12 of 21 candidates were matched with differentially expressed genes in the DN transcriptomic data. By providing a novel interaction network and prediction, GCNet contributes to improving the understanding of normal glomerular function and will be useful for detecting target cytoskeleton molecules of interest that may be involved in glomerular diseases in future studies.

## Introduction

Glomerular diseases account for a large proportion of chronic kidney diseases (CKDs) and are becoming an important health problem[1]. Glomerular diseases are mainly caused by structural abnormalities and dysfunction of the glomerular filtration barrier, which consists of the fenestrated endothelium, basement membrane and glomerular visceral epithelial cells, also termed podocytes. This filtration barrier function as like a complex biological sieve that dynamically controls the filtering of large volumes of plasma while retaining most proteins within the circulation[2, 3]. To withstand the high pressure in the capillaries and maintain precise filtration properties, glomerular cells (especially podocytes) appropriately adapt their morphologies and regulate their shapes. Because morphology and cell shape are mainly regulated by dynamic, interconnected actin and microtubule polymers, the cytoskeleton plays an essential role in maintaining the normal function of the glomerulus[4]. Previous elegant studies have revealed that many cytoskeleton proteins (e.g., actinin 4, synaptopodin, actin-related protein 2/3 complex, cofilin, and myosin IE) play roles in glomerular diseases[5–9]. For example, a mutation in *ACTN4*, which encodes actinin 4, causes hereditary focal segmental glomerulosclerosis (FSGS). In FSGS patients, changes in podocyte morphology and dysfunction of glomerular filtration are observed, resulting in clinical signs of proteinuria and “foot process effacement” detected via electron microscopy[8]. In addition to the key role of the podocyte cytoskeleton in the progression of glomerular diseases, variations in mesangial and endothelial cells also play roles[10–12]. However, most relevant studies have examined a single cytoskeleton gene, and few have addressed the cytoskeleton network or performed systematic analyses of the interactions among cytoskeleton proteins. GlomNet and PodNet were previously established by using bioinformatics tools to analyze protein-protein interaction network to increase understanding of renal disease pathophysiology[13, 14]. Refining the network with regard to specific cellular component or other limited conditions will increase its utility for the prediction of new key genes/proteins in experimental studies. Based on the continuous advances in bioinformatics and omics technologies, we generated a collection of glomerular cytoskeleton proteins and then constructed a protein-protein interaction network, designated the Glomerular Cytoskeleton Network (GCNet). Moreover, the components of GCNet were evaluated in association with various renal diseases, and 21 novel potential key cytoskeleton were highlighted. Our study not only provides new insights into the understanding of mechanisms underlying glomerular diseases but also enables the prediction of key regulators involved in glomerular diseases.

## Results

### Identification of all human cytoskeleton genes/proteins based on Gene Ontology terms and annotations

As the foundation for establishing the network of renal glomerular cytoskeleton proteins, we first identified all known cytoskeleton genes. The cytoskeleton is a network of filamentous elements composed of various proteins contained within the cytoplasm. The cytoskeleton of mammalian cells has traditionally been considered to include three major components: microfilaments, microtubules and intermediate filaments. Due to increased understanding of the function and structure of the cytoskeleton acquired over the years, its definition has been expanded to include elements such as the microtrabecular lattice, septins, cross-linkers, capping proteins and other structures characterized by a polymeric filamentous nature and a long-range order within cells[15–18]. To identify all cytoskeleton genes/proteins, annotated human cytoskeleton proteins and their descendants were retrieved from the GO database. After removing unannotated and repeated proteins, a total of 2030 cytoskeleton genes remained and were used as the foundation for our study (S1 Table).

### Collection of renal glomerulus-enriched genes

To identify genes enriched in the renal glomerulus, nine available glomerular expression datasets were examined. These datasets included two human SAGE profiles, two human Affymetrix array profiles, one human Stanford cDNA microarray profile, one human plasmid library, one mouse cDNA microarray profile, one mouse EST library, and one mouse Affymetrix array profile. The genes enriched in the mouse glomerulus were mapped to human Entrez genes and then selected for analysis. As shown in Table 1, the nine different expression datasets were summarized and compared [13, 19–26]. In summary, a total of 2929 genes were found to be enriched in the renal glomerulus through at least one profiling method (S2 Table).

**Table 1:** Summary of the characteristics of the nine expression datasets.

Researchers	Year	Method	Species	Glomerulus-Enriched Genes (Human)	Number and Proportion of GO Cytoskeleton	Selection Criteria	Reference
Chabardes-Garonne et al	2003	SAGE profiling	Human	148	32/148 (21.62%)	*P* < 0.01, seven-fold or more difference with at least three nephron libraries.	[19]
Higgins et al	2004	Stanford cDNA microarray profiling	Human	102	28/102 (17.64%)	Cluster analysis, genes predominantly expressed in glomeruli than others	[20]
Takemoto et al	2006	GlomChip profiling	Mouse	310	53/310 (17.10%)	*P* < 0.05, more than two-fold difference with nonglomerular kidney tissue	[21]
He et al	2007	EST libraries Comparison	Mouse	165	32/165 (19.39%)	*P* < 0.05, more than three-fold difference with whole kidney tissue	[22]
He et al	2008	Affymetrix profiling	Mouse	914	118/914 (12.91%)	*P* < 0.05, more than two-fold difference with nonglomerular kidney tissue	[13]
Nyström et al	2009	SAGE profiling	Human	130	25/130 (19.23%)	Comparison to pooled SAGE libraries for non-glomerular tissues and cells	[24]
Cuellar et al	2009	cDNA library (Plasmid cloning)	Human	202	23/202 (11.39%)	Sequence analysis and comparison with UniGene database	[23]
Lindenmeyer et al	2010	Affymetrix profiling	Human	675	138/675 (20.44%)	*P* < 0.05, Comparison of isolated glomeruli with tubulointerstitial compartment from biopsies of living donors	[25]
Woroniecka et al	2011	Affymetrix profiling	Human	1890	299/1890 (15.82%)	*P* < 0.05, and fold change>1.5. Comparison of isolated glomeruli with tubulointerstitial compartment from biopsies of living donors	[26]

### Generation of the glomerular cytoskeleton protein-protein interaction network

**Fig 1:**
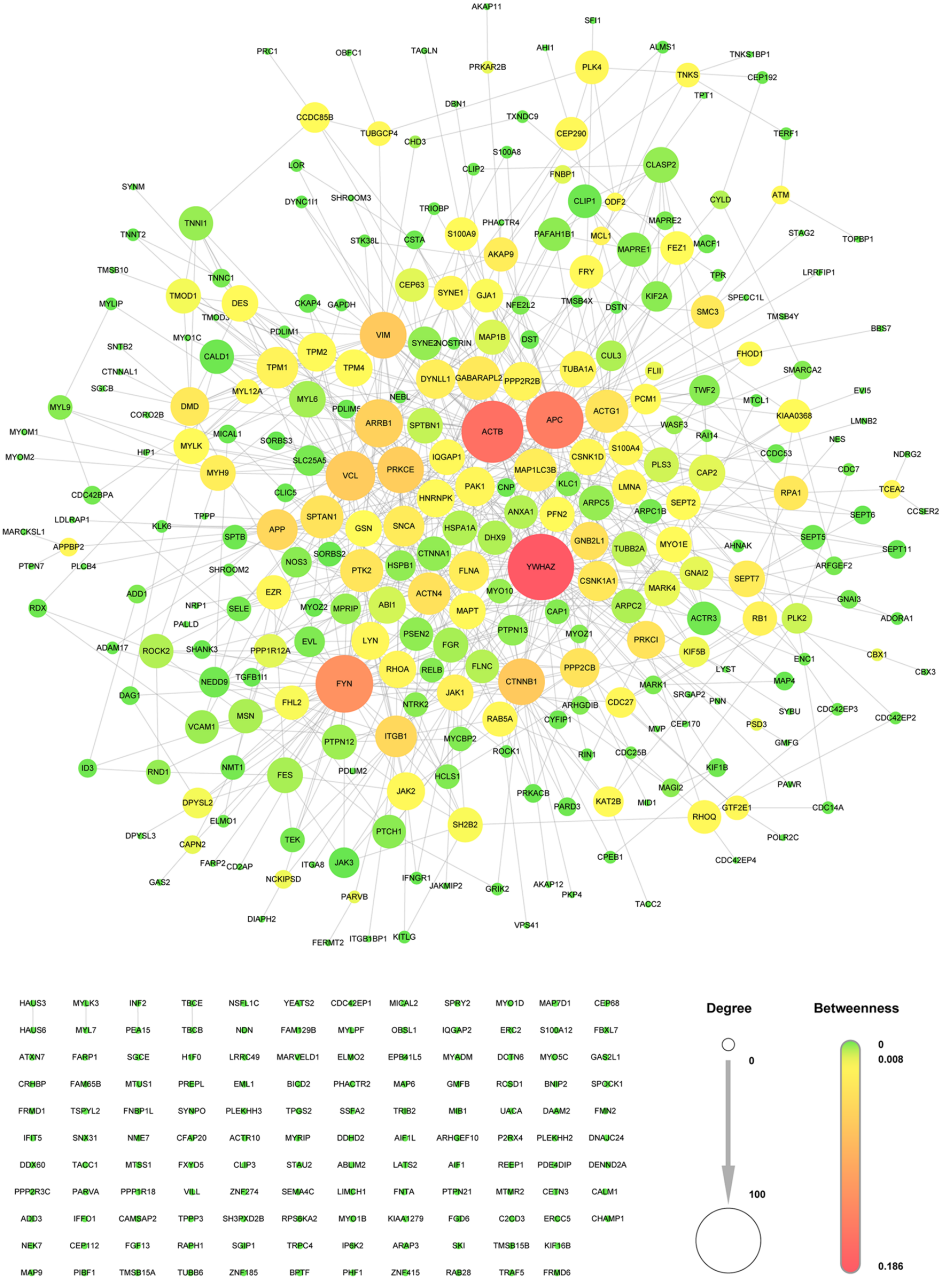
Glomerular Cytoskeleton Network. The nodes represent proteins (labeled with the gene name), and the edges represent the interactions of the corresponding proteins. The importance of each node can be differentiated by its size (degree) and color (betweenness centrality). The larger the size of a node, the more interactions it exhibits; in addition the closer its color is to red, the greater is its betweenness centrality.

Cytoskeleton genes enriched in the glomerulus were extracted from two well-established public databases (HPRD and STRING) (S3 Table) to build the GCNet based on protein-protein interactions[27, 28]. The resultant network (Fig 1, all nodes and edges are listed in S4 Table) consisted of 426 proteins (nodes labeled by gene name) and 827 PPI interactions (edges). The largest connected component included approximately 70% of the nodes and 99% of the edges, with a mean of 1.9 interactions per node. With the exception of several two-node islets, the remaining 122 nodes were disconnected from one another.

### Comparison of GCNet with non-glomerular dataset

It is well known that podocytes share some common characteristics with other cells such as neurons and smooth muscle cells. Several proteins that play important roles in other tissues were also found to be involved in function of podocytes. The most representative is *SYNPO*. To make a potential clue to further understand the function of glomerular enriched cytoskeleton, we compared genes in GCNet with neuronal related genes and genes mainly expressed in smooth muscle cells as well as in muscle and heart. The data on neuronal/brain-associated genes and smooth muscle-related genes was derived from an elegant study performed by *Maja T et al* by using Digital Differential Display (DDD) analysis. The results revealed that there were 71 of 414, 18 of 89, and 63 of 159 cytoskeleton genes in neuronal related, smooth muscle cell related, and muscle and heart related gene, respectively. And also 19 of 71, 8 of 18, and 24 of 63 glomerular cytoskeleton genes were overlapped with neuronal related, smooth muscle cell related, and muscle and heart related gene, respectively (S5 Table). In addition, 3 of 21 candidates, *MYL9*, *PDLIM5*, and *PKP4* were included among these overlapping genes, indicating that these genes were both involved in glomerular and other tissues’ function.

### Prediction of the key glomerular cytoskeleton components and generation of the predicted Disease-related Glomerular Cytoskeleton Network

Genes that were differentially expressed (differentially expressed genes were defined with a p-value of < 0.05 between the diseases group and healthy group) between glomeruli isolated from FSGS, Membranous Nephropathy (MN), Minimal Change Disease (MCD), Diabetic Nephropathy (DN), and IgA nephropathy patients versus those from healthy living donors were identified based on the study conducted by Ju *et al*. in Nephromine (original expression data were published in reference 29 and uploaded to Gene Expression Omnibus) [29]. Table 2 shows the number of differentially expressed genes included in GCNet, as confirmed by the presence of proteinuria disease.

**Table 2:** Summary of the characteristics of GCNet in the five glomerular diseases.

Disease	Number of Up-regulated gene	Number of GCNet embraced(up)	Number of down-regulated gene	Number of GCNet embraced(down)	Number of total DEGs GCNet embraced(% in GCNet)
**FSGS**	1859	76	2820	83	159(37.3%)
**MN**	4755	134	2296	78	212(49.8%)
**MCD**	994	18	1282	89	107(25.1%)
**DN**	3781	103	5447	160	263(61.7%)
**IgA**	5637	94	3812	178	272(63.8%)

(Abbreviation: DEGs = differentially expressed genes).

Further, 361 differentially expressed cytoskeleton genes were identified in association with at least one renal disease, in addition to 37 that were associated with all five renal diseases (S6 Table). The characteristics of all of the differentially expressed cytoskeleton genes are listed in Table 3. Furthermore, 21 of the 37 genes were consistently differentially expressed in all five glomerular diseases, 3 of which were consistently up-regulated, and 18 of which consistently down-regulated.

**Table 3:** Consistently regulated genes in GCNet.

Differentially expressed in	Direction consistent		Direction inconsistent	Total
	Up-regulated	Down-regulated		
**None**	\	\	\	65
**1 disease**	23	31	\	54
**2 diseases**	23	39	44	106
**3 diseases**	18	23	53	94
**4 diseases**	15	17	38	70
**All diseases**	3	18	16	37

**Fig 2:**
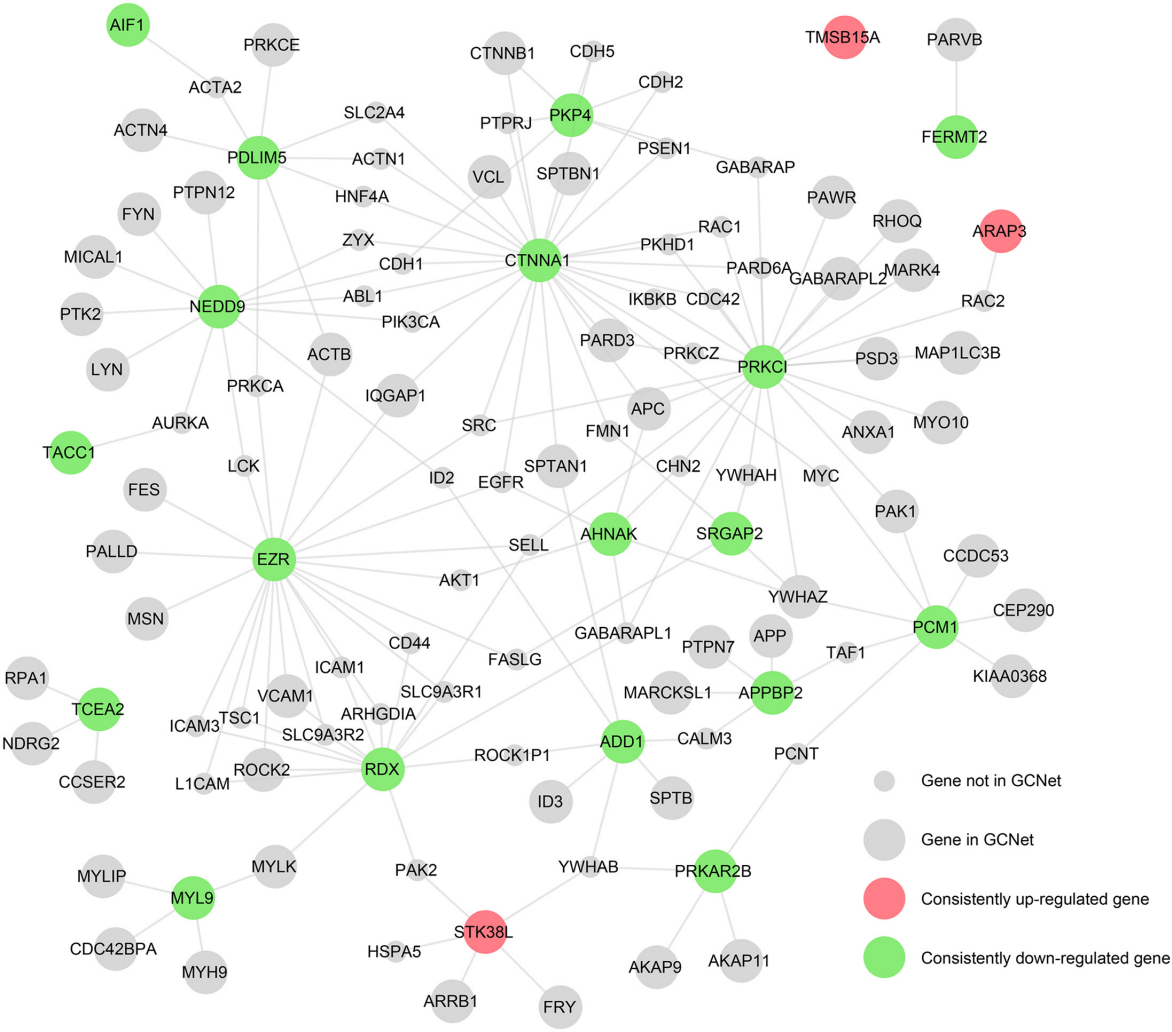
Predicted Disease-related Glomerular Cytoskeleton Network. The 3 up-regulated genes detected in all 5 diseases are colored red, and the 18 down-regulated genes are colored green. The large-sized nodes represent Glomerular Cytoskeleton Network genes, and the small-sized nodes represent those that are not in the Glomerular Cytoskeleton Network gene list but that link the key genes together.

The consistent regulatory patterns of these 21 genes in the 5 glomerular diseases suggested that they were common potential candidates that may play key roles in glomerular function. To understand the roles of these cytoskeleton elements in greater detail, the predicted Disease-related Glomerular Cytoskeleton Network (pDGCNet) was established based on the GCNet (Fig 2) that more clearly showed the protein-protein interactions with other cytoskeleton components.

### General description of candidates key cytoskeleton components involved in glomerular diseases

**Fig 3:**
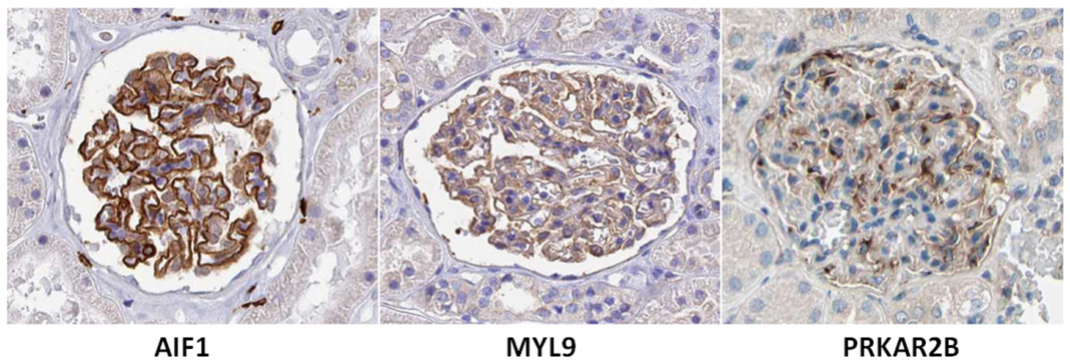
Validation of potential candidates based on Human Protein Atlas (HPA) staining images. High-throughput immunohistochemical (IHC) stainings images from HPA (http://www.proteinatlas.org) were used to validate the protein expression of potential candidates, among which allograft inflammatory factor 1 (*AIF1*), myosin regulatory light chain 9 (*MYL9*) and protein kinase cAMP-dependent regulatory type II beta (*PRKAR2B*) were found to be only expressed in the glomeruli but not in the tubules.

The mRNA levels of 21 candidates key cytoskeleton components were previously identified. To examine the protein expression of these candidates, the Immunohistochemistry (IHC) staining data were extracted from the Human Protein Atlas (HPA) (http://www.proteinatlas.org). Of the 21 candidates, 19 included data for evaluation of protein expression (HPA Version: 13 2014-11-06). Of these 19 proteins, 17(89.5%) were expressed in the glomeruli and 3 were only expressed in the glomeruli but not in the tubules. These proteins included allograft inflammatory factor 1 (*AIF1*), myosin regulatory light chain 9 (*MYL9*) and protein kinase cAMP-dependent regulatory type II beta (*PRKAR2B*) and IHC staining images from HPA are shown in Fig 3.

**Fig 4:**
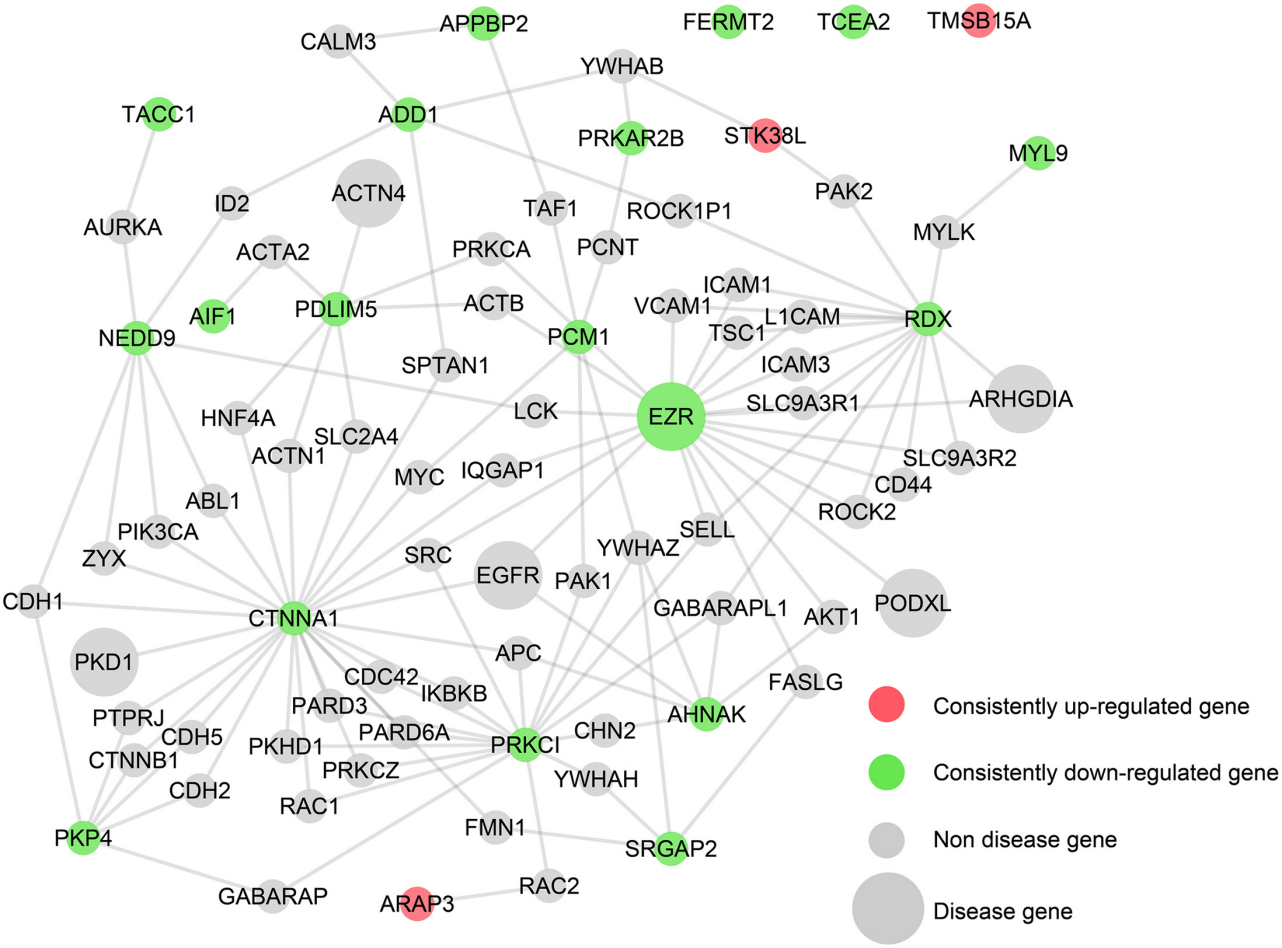
Protein protein interactions between the potential candidates and known glomerular disease genes. Protein–protein interaction network for 21 candidate proteins (labeled by gene name). Three up-regulated genes in all 5 diseases are colored red, and 18 down-regulated genes are colored green. Proteins are represented by red (up-regulated) and green (down-regulated) nodes, and the known glomerular disease genes selected from the OMIM database are presented in a larger size.

After validation of the expression of these candidates at both the mRNA and protein levels, functional analysis was also performed. Known glomerular disease genes (S7 Table) were retrieved from the Online Mendelian Inheritance in Man (OMIM) database, which includes genes whose mutations cause glomerular abnormalities in humans, such as *NPHS1*, *ACTN4* and *ARHGDIA*. The protein-protein interactions between the candidates and the known glomerular disease genes were mapped using HPRD and STRING to deduce possible functions and interactions (Fig 4). As shown in Fig 4, these candidates not only interacted with the widely known glomerular proteins, including interactions between *PDLIM5-ACTN4*, and *RDX-ARHGDIA*, but they also interacted with several predicted proteins, such as *CDC42* and *RAC1*, which have already been proven to play a key role in the podocyte function. In addition, on the basis of known glomerular disease genes-phenotype data from the OMIM database, we used our previously published algorithm, CIPHER, to predict and prioritize disease genes to determine whether these candidates have a strong associations with the known glomerular diseases[30]. This assessment allowed for the prediction of genotype-phenotype correlations.

**Fig 5:**
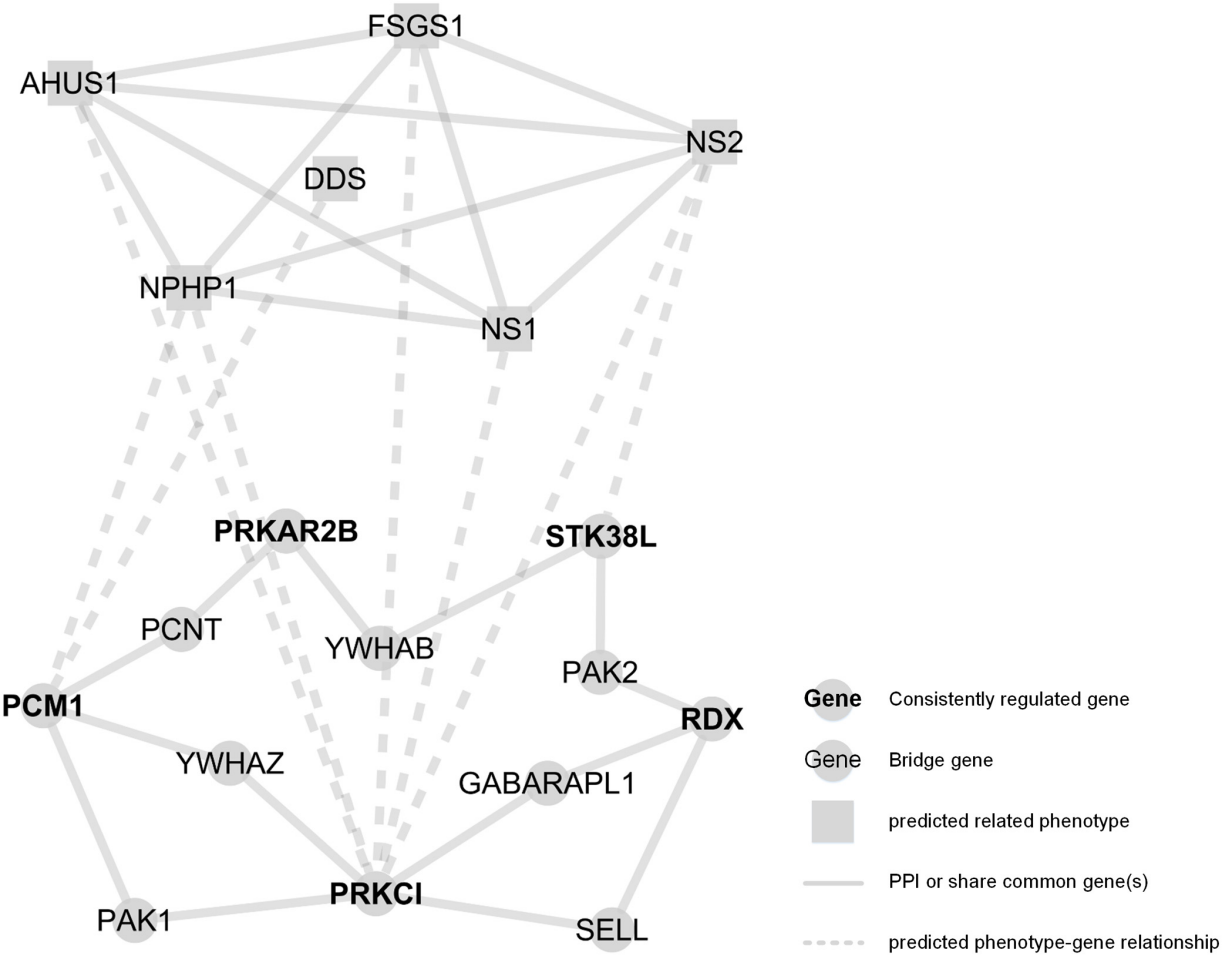
Predicted diseases associated with the potential candidates. A total of 6 diseases (square) related to 3 genes (circle) are shown. Diseases linked together share at least one common disease gene; genes are connected by the shortest path in Fig 3 (*AHUS1*, HEMOLYTIC UREMIC SYNDROME, ATYPICAL, SUSCEPTIBILITY TO, 1, mutation of *CFH*; DDS, DENYS-DRASH SYNDROME, mutation of *WT1*; FSGS1, FOCAL SEGMENTAL GLOMERULOSCLEROSIS 1, mutation of *ACTN4*; NPHP1, NEPHRONOPHTHISIS 1, mutation of *NPHP1*; NS1, NEPHROTIC SYNDROME, TYPE 1, mutation of *NPHS1*; NS2, NEPHROTIC SYNDROME, TYPE 2, mutation of *NPHS2*).

As shown in Fig 5, 3 candidates were predicted to be directly related to the known glomerular diseases, with associations detected between *PRKCI* and Nephrotic Syndrome types 1 and 2 and FSGS1, providing valuable insights into the key molecules involved in glomerular diseases. All of these predictions are listed in S8 Table.

### Validation of predicted candidates by real-time quantitative PCR and comparison with existed transcriptomic data

**Fig 6:**
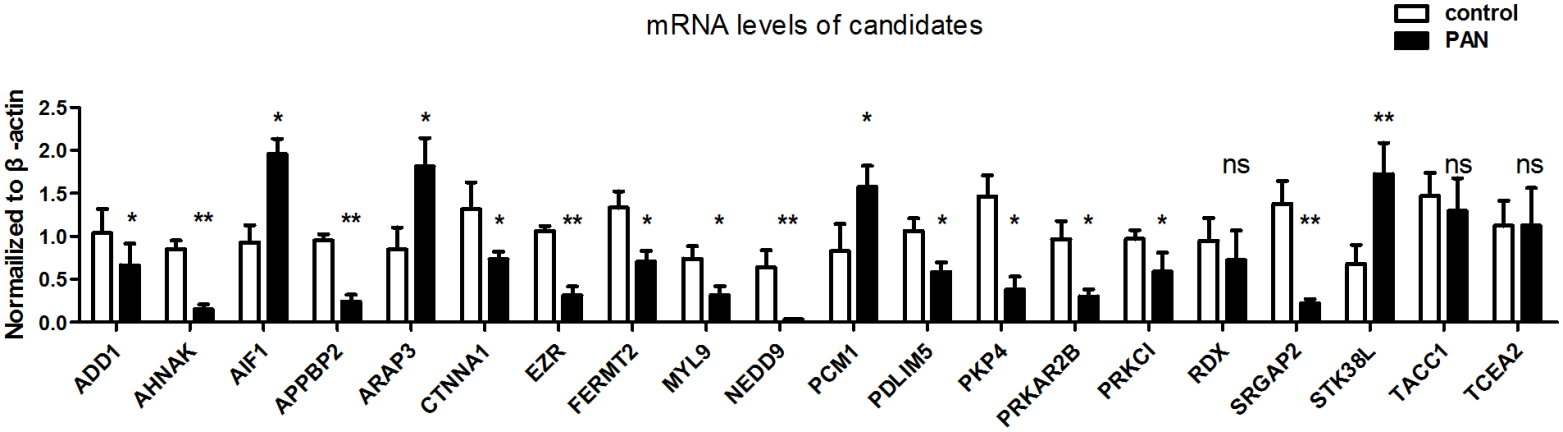
Candidate mRNA levels in glomeruli isolated from a PAN induced rat nephropathy model. Except for *TMSB15A*, the mRNA levels of the other 20 candidates were examined by real-time quantitative PCR. The mRNA levels of 13 candidates were down-regulated, those of 4 candidates were up-regulated, and those of 3 candidates were not statistical significance in the PAN group compared with the control group. (*p, 0.05 vs. control; **p, 0.01 vs. control; ns: no statistical significance; n = 5. PAN: puromycin aminonucleoside).

To further validate all 21 predicted candidates, we performed real-time quantitative PCR on glomeruli isolated from a puromycin aminonucleoside (PAN) induced rat model. Due to the rat mRNA sequence of candidate *TMSB15A* could not be acquired in public database; thus, we examined the mRNA levels of the other 20 candidates. As shown in Fig 6, 17 of 20 (85%) candidates were significantly regulated in the PAN treatment group compared with the control group. 15 of 17 candidates’ regulation directions were consistent with the results for the 5 glomerular diseases. There are *ADD1*, *AHNAK*, *APPBP2*, *ARAP3*, *CTNNA1*, *EZR*, *FERMT2*, *MYL9*, *NEDD9*, *PDLIM5*, *PKP4*, *PRKAR2B*, *PRKCI*, and *STK38L*. These results suggested that these genes were involved in PAN-induced nephropathy. They may play an important role in glomerular diseases. Also, we have matched the differential gene expression of 21 candidates with data from a transcriptomic study of DN published by *Woroniecka et al*, which also included the glomerular enriched dataset used in our study to select the glomerular enriched genes[26]. Surprisingly, 12 of 21 candidates (over 50%) were also identified as differentially expressed genes in diseased and healthy kidney glomeruli. There are *MYL9*, *PKP4*, *NEDD9*, *PCM1*, *APPBP2*, *PRKAR2B*, *RDX*, *FERMT2*, *AIF1*, *AHNAK*, *PDLIM5*, and *SRGAP2*. In addition, all of these candidates’ regulation directions (up or down regulated) were consistent with those in DN transcriptomic dataset. Objectively speaking, the high match rate was partly due to the fact that a proportion of glomerular enriched cytoskeleton was included in this study. However, only 3 of 12 these genes were alone extracted from this study, another 9 of 12 candidates were extracted from other datasets or from at least 2 datasets included the dataset in this study. These results reveal that our predicted candidates have potentially important roles in glomerular diseases.

## Methods

### Sources of human cytoskeleton genes/proteins

To obtain the most comprehensive information on the cytoskeleton as possible, we downloaded the lists of proteins (labeled by gene name) under the following three related GO terms from QuickGO (http://www.ebi.ac.uk/QuickGO) in Sep. 2014: "cytoskeleton" (GO: 0005856), "microtubule binding" (GO: 0008017) and "actin binding" (GO: 0003779). We used the combination of these three sets, which contained 2030 genes, in subsequent analyses (S1 Table).

### Sources of renal glomerulus-enriched genes

We manually examined the renal glomerulus-related literature published during from 2003 to the present. We selected reported gene expression profiles from microdissected glomeruli and generated the most comprehensive dataset of human renal glomerulus-enriched genes (the selection criteria for the “enriched” genes were the same as those used in the original research and are listed in Table 1) produced to date, to the best of our knowledge. We chose a total of 9 relevant profiles, including two human SAGE profiles, two human Affymetrix array profiles, one human Stanford cDNA microarray profile, one human plasmid library, one mouse cDNA microarray profile, one mouse EST library, and one mouse Affymetrix array profile. All of the mouse gene identifiers were converted into human identifiers with Biomart (http://www.biomart.org/). In summary, a total of 2929 genes were found to be enriched in the renal glomerulus through at least one profiling method (S2 Table).

### Generation of the Glomerular Cytoskeleton Network

To include as many credible protein-protein interactions as possible, we integrated protein-protein interaction information from two well-established public databases, HPRD [27] and STRING[28] (S3 Table). The confidence scores of STRING database which are derived by benchmarking the performance of the predictions against a common reference set of trusted, true associations, were used in our study to include the more useful predicted associations[31]. STRING preprocessed each data source to get the confidence scores (strong stringency data sources, such as co-IP and yeast 2 hybrid, usually obtain higher scores), which were integrated to produce a combined score for each PPI which works as the stringency of the interactions identified. According to the formula for calculating the integrated score, interactions supported by a single data source could be scored 0.9 at most. Thus, we chose 0.9 as our threshold to extract the PPIs at least identified by one strong stringency source at least. We used the links between the nodes, which corresponded to overlaps of human cytoskeletal genes and renal glomerulus-enriched genes, to construct the GCNet. All nodes and edges are listed in S4 Table.

To visualize the resulting network and to obtain an intuitive perspective, we performed two measurements employed in graph theory (degree and betweenness) to determine the importance of each node (gene). The degree (represented by the node size) of a vertex, *ν*, is the number of vertices that are linked to it. The betweenness (represented by the node color) of a vertex, *ν*, can be roughly regarded as the number of times that it is present in the shortest path between two other nodes, and it can be more precisely denoted by

$${\text{C}}_{\text{B}}(v)=\sum_{v\neq s\neq t\in V}\frac{\sigma_{st}(v)}{\sigma_{st}}$$

where *σ*_*st*_ represents the number of existing shortest paths between node *s* and node *t*, and *σ*_*st*_ *(v)* represents the number of paths that pass through node *ν*.

### Generation of the predicted Disease-related Glomerular Cytoskeleton Network

Differentially expressed genes identified in FSGS, MN, MCD, DN, and IgA patients were downloaded from the *Nephromine* website (http://www.nephromine.org/). We checked the expression patterns of the genes in the GCNet in FSGS, MN, MCD, DN, and IgA contexts. We selected the differentially expressed genes with a p-value of < 0.05 (S6 Table) and identified 21 consistently regulated genes that also appeared in the GCNet. Because the pairs of these 21 genes were barely connected in our integrated PPI network, we further introduced some of their common first-order neighbors as “bridge” nodes to reconstruct a subnetwork. We designated this core subnetwork as pDGCNet, which is a major part of the GCNet and is strongly related to renal diseases.

### Evaluation of protein expression in HPA

We evaluated the protein expression of 21 potential candidate key cytoskeletal components based on HPA Version 13.0–2014.11.06. The following criteria were used to distinguish glomerular-expressed proteins: 1) proteins that were annotated to the glomeruli according to the criteria of the HPA; and 2) proteins whose expression was not confirmed using different HPA antibodies (not annotated) but that were shown to be expressed in the glomeruli and consistently in different kidney tissues by IHC staining.

### Sources of known glomerular disease genes

Known glomerular disease genes were acquired from the OMIM database. “Glomerulus” was used as the key search word, and 333 items were retrieved. Relevant genes associated with each of the items were selected according to specific inclusion and exclusion criteria. The inclusion criteria were as follows: 1) genes for which a mutation causes glomerular injury; 2) genes for which a mutation causes a syndrome including glomerular injury; 3) genes for which a mutation does not cause glomerular injury, but relevant genetically modified animal models have shown glomerular injury; 4) genes and encoded proteins reported to be involved in glomerular injury based on *in vivo* and/or *in vitro* studies; and 5) genes and encoded proteins reported to be involved in glomerular development. The exclusion criteria were as follows: 1) genes for which a mutation causes tubular injury, but not glomerular injury; 2) genes for which a mutation causes renal function abnormality, but there are a lack of studies exploring whether glomerular injury is present; and 3) genes that are expressed in the glomerulus for which there are a lack of studies exploring potential involvement in glomerular injury. Manual selection of genes according to these criteria resulted in the identification of 125 known glomerular disease genes (S7 Table).

### Identification of topological relationships between the potential candidates and glomerular disease genes

To identify the topological relationships between the potential candidates and known glomerular disease genes, we introduced a few genes as bridge nodes to construct a connected graph, similar to the generation of the disease glomerular cytoskeleton network.

### Prediction of the genotype-phenotype correlations between the potential candidates and known glomerular diseases

To determine whether the potential candidates have strong associations with the known glomerular diseases, we employed our previously published algorithm, CIPHER, which takes the similarities between phenotypes and the topological distances between genes in the protein network into consideration in prioritization of the genes responsible for the specific diseases. For each phenotype, we regarded the top 1000 genes as being highly likely to be involved in genotype-phenotype correlations with the associated disease. All of the predicted relationships and specific rankings are listed in S8 Table.

### Animal model

Sprague-Dawley Male rats (n = 10, 6–8 weeks old) were purchased from the Animal Center of Peking University Health Science Center. The animal studies were approved by the Animal Research Review Board of Peking University First Hospital (No.J201306). The rats were randomly divided into two groups. The first group (n = 5) was treated with normal saline, and the second group (n = 5) received a single intraperitoneal injection of PAN at 150 mg/kg body weight (Sigma-Aldrich, USA). All of the rats were sacrificed on day 10, and the kidneys were removed. One kidney was used to isolate glomeruli using the differential sieving method[32].

### Real-time quantitative PCR

Total RNA was extracted using TRIzol reagent (Invitrogen, USA) according to the manufacturer’s instructions. RNA concentration and purity were assessed using a NanoDrop 2000 Spectrophotometer (Thermo Scientific, USA). In total, 2 mg of RNA was reverse transcribed using the high-capacity cDNA Reverse Transcriptase (RT) Kit (Invitrogen, USA) according to the manufacturer’s protocol. The real-time quantitative PCR mixture contained 2.0 μl of RT product, 10 μl of SYBR Green PCR mix (TransGen Biotech, China) and 0.5 mM primers in a final volume of 20 μl. The primers used in real-time PCR are listed S9 Table.

## Discussion

In the present study, the GCNet was established to explore potential novel and important genes/proteins involved in glomerular diseases. Information on the cytoskeleton genes/proteins was retrieved from the GO public database. Cytoskeleton, as per the traditional definition, contains only microfilaments, microtubules and intermediate filaments. However, the actin-based foot process of podocytes, with its highly dynamic properties, is the functional unit of the filtration barrier and is extensively regulated by many actin-binding proteins, such as the Rho GTPase family proteins[33], synaptopodin[9], and Nck[34]. The narrow traditional definition of the cytoskeleton does not include certain potential key interactions in renal diseases. Hence, both the cytoskeleton and its descendants were examined in this study to search for useful information more broadly.

In the kidney glomerulus, the glomerular capillaries, combined with the glomerular basement membrane (GBM), provide stable support for the tuft. A large amount of experimental data and clinical knowledge indicate that podocytes play key roles in glomerular diseases and that different glomerular cells play various specific roles and interact with each other in the maintenance of normal structure and function. For example, smooth muscle-like mesangial cells located inside of the GBM act as structural supporters and connectors through their contractile activity and secretory function. Moreover, several expression profiling studies have focused on the glomerulus and have identified glomerular-enriched genes to achieve a systematic understanding of the development and function of this structure and its derangement in glomerular diseases[25, 26]. In the present study, nine available glomerular expression profiles obtained using six different methods identified 2929 genes that were enriched in the glomerulus. Due to the different technical platforms, different selection criteria, and different experimental protocols and normalization methods used, few common genes were found among these profiles. Similar issues have been reported previously by HE *et al*. and Lindenmeyer *et al*. [22, 25]. To obtain the most comprehensive glomerular set, we combined the gene lists from the nine profiles. Also, podocytes are an inherent cell type in the glomerulus. Not only genetic studies have clearly demonstrated that mutation of podocyte cytoskeleton gene can cause renal disease, leading to rearrangement of the actin cytoskeleton and ultimately to glomerular failure, but also a lot of animal model studies have demonstrated that podocyte cytoskeleton genes play a very important role in glomerular diseases. Thus, we have also noted which glomerular enriched genes were also enriched in podocytes in GCNet genes list (S4 Table), as determined according to *Melanie Boerries et al* study which identified the podocyte-enriched genes by using Affymetrix MoGene 1.0 ST chips [35]. As a result, there are 213 of 426 glomerular enriched cytoskeletal genes also enriched in podocytes.

Knowledge of protein-protein interactions has rapidly accumulated over the last decade. However, the reliability and reproducibility of the PPI data are becoming increasingly important issues encountered by researchers. To address these challenges, we constructed GCNet based on both literature and experimental validation, and we expanded the network of protein-protein interactions with caution. For example, we took advantage of the protein-protein associations available in two well-established public databases, HPRD and STRING; these associations have been proven to be useful in a variety of systematic and large-scale studies [27, 28]. However, we acknowledge that a portion of the current PPI information was derived from *in vitro* experiments; therefore the network that we constructed in a renal cytoskeleton context is expected to contain a few false-positive PPIs, which makes the connectivity observed in the resulting network slightly over-optimistic.

The main aim of the GCNet is to contribute to further identification of the potential key genes and proteins involved in common glomerular diseases to facilitate analysis of the possible underlying molecular mechanisms. Therefore, the GCNet was applied to examine several microarray datasets derived from proteinuric renal disease, and pDGCNet was consequently developed. As shown in Fig 2, we only included 21 consistently regulated genes in pDGCNet to figure out the relationships between the 21 candidates with other cytoskeleton components. Actually, a lot of others cytoskeleton which were also regulated in glomerular diseases, but those that were not regulated or were not consistent regulated in all 5 diseases were excluded as candidates. As shown in Fig 1, *YWHAZ*, *ACTB*, *APC*, and *FYN* were the most important genes involved in GCNet, it means these genes were tightly connected with other cytoskeleton components. However, these genes were not included among the 21 consistently regulated genes (Fig 2). We checked the most important 4 genes *YWHAZ*, *ACTB*, *APC*, and *FYN* in differentially regulated genes list (S6 Table) and found that they were all regulated in at least 3 glomerular diseases, especially the *APC* was regulated in all 5 glomerular diseases. But it was not consistently regulated among these diseases, as it was, down-regulated in 4 and up-regulated in 1. According to our criterion that the direction of regulation should be consistent among all 5 diseases, *APC* was excluded from the 21 candidates. Several high-throughput microarray studies have been performed on patients and animal models to identify differentially expressed genes and to deepen the understanding of disease mechanisms. However, different studies have adopted different platforms, criteria, normalization methods, etc. To minimize variations among studies, we focused our analysis on expression data reported by the same group(*Ju et al*); these data include batch-corrected expression data derived from FSGS, MCD, MN, IgA and DN patients[29]. Both the up- and down-regulated genes were listed in that study. All five renal diseases are characterized by glomerular abnormalities and varying degrees of proteinuria, which have been proven to be closely associated with the abnormal function of the cytoskeleton in glomeruli, especially in podocytes. All 21 candidates were commonly regulated in all 5 glomerular diseases, indicating that these candidates may be involved in a common mechanism of glomerular diseases. Also, there are distinct mechanisms in these 5 different diseases. As shown in S6 Table, some of the genes were inconsistently regulated among the different glomerular diseases, indicating that there are also distinct mechanisms among the different glomerular diseases, thereby leading to inconsistently regulated direction for the same gene. These genes may have different roles in the different diseases. Also, the data acquired from the microarrays were limited to mRNA expression data and did not provide information on the expression of proteins, which are regulated by posttranslational modifications, such as phosphorylation; thus, these data did not provide insights into physiological performance. Therefore, the 21 selected candidates were limited to commonly dysregulated genes involved in signaling pathways. This is the reason why the known cytoskeletal regulators of glomerular diseases, such as synaptopodin and actinin 4, were not included among our candidates. Nevertheless, dysregulation of the expression of the candidates was associated with that of known genes/proteins, such as *ACTN4*, *PKD1*, and *ARHGDIA* (Fig 4). And also, one of the 21 candidates, *EZR* has been previously demonstrated to play very important roles in many glomerular diseases. *Wasik AA et al* have reported that ezrin is down-regulated in diabetic kidney glomeruli and that it regulates actin reorganization and glucose uptake via *GLUT1* in cultured podocytes[36]. Also, in *Scott J*. *Harvey et al* study, ezrin is regulated in a mouse model with a podocyte-specific deletion of dicer, which alters cytoskeletal dynamics and causes glomerular disease[37]. Ezrin also combines with podocalyxin to form a complex that interacts with the actin cytoskeleton. Disruption of this complex has been shown to cause a dramatic loss of foot processes in PAN, protamine sulfate, or sialidase-treated rats by *Tetsuro Takeda et al*[38]. It is also important to figure out early signaling events to late. Datasets used in our study were limited to settled stage. Because of the lack of the datasets on each glomerular diseases stages, the predicted candidate genes in this study were not been varied from the early to late stage.

To facilitate further study and detection of cytoskeleton of interest, two strategies can be used. The first is detection of cytoskeleton of interest. If expression of one cytoskeleton is known to be correlated with that of well-known genes or if that cytoskeleton is differentially expressed in a subset of proteinuria diseases, then, it can first be examined whether it is were enriched in glomeruli or podocytes (S2 and S4 Tables). Then, the interactions between other cytoskeleton can be searched for in Fig 1 and S4 Table to determine whether they are related to known functional cytoskeleton. Next, the interested cytoskeleton can be examined with known glomerular diseases genes to see the interaction between them (Fig 4 and S7 Table). To examine the regulation of cytoskeleton of interest in the established dataset of proteinuria diseases, these genes can be located in S6 Table to clearly determine whether they are up- or down- regulated in the five known proteinuria diseases (FSGS, MN, MCD, DN, and IgA). The second strategy is to choose one of 21 candidates predicted in present study. A total of 20 of the 21 candidates have been shown to be expressed in glomeruli at mRNA levels in present study (Fig 6). In addition, 17 of the 21 candidates have been demonstrated to be expressed in glomeruli at protein levels by HPA. The 21 candidates were consistently regulated among all five proteinuria diseases and have high probabilities of involvement in glomerular diseases. And also, the regulation of 17 of the 21 candidates was verified in PAN-induced rat model (Fig 6).

## Supporting Information

S1 Table Human cytoskeleton genes identified by Gene Ontology analysis. (XLS)

S2 Table Glomerulus-enriched genes from nine available glomerular expression profiles. (XLS)

S3 Table Protein-protein interactions in the network integrated from HPRD and STRING. (XLS)

S4 Table All nodes and edges in the GCNet. (XLS)

S5 Table Neuron/brain-associated, smooth muscle cell associated, and muscle- and heart associated gene list. Genes marked as “Yes” are present in GCNet.
(XLS)

S6 Table Differentially expressed GCNet genes in five glomerular diseases. (XLS)

S7 Table Known glomerular disease genes from OMIM. (XLS)

S8 Table Known glomerular diseases related to the candidates predicted by CIPHER. (XLS)

S9 Table The primers used in real-time PCR. (XLS)
